# Amyotrophic Lateral Sclerosis Mimic Syndrome in a 24-Year-Old Man with Chiari 1 Malformation and Syringomyelia: A Clinical Case

**DOI:** 10.3390/jcm12082932

**Published:** 2023-04-18

**Authors:** Mustafa Al-Zamil, Natalia A. Shnayder, Tatiana K. Davydova, Regina F. Nasyrova, Vera V. Trefilova, Ekaterina A. Narodova, Marina M. Petrova, Irina V. Romanova, Galina A. Chumakova

**Affiliations:** 1 Department of Physiotherapy, Faculty of Continuing Medical Education, Peoples’ Friendship University of Russia, 117198 Moscow, Russia; 2 Institute of Personalized Psychiatry and Neurology, V.M. Bekhterev National Medical Research Centre for Psychiatry and Neurology, 192019 Saint Petersburg, Russia; nreginaf77@gmail.com; 3 Shared Core Facilities “Molecular and Cell Technologies”, V.F. Voino-Yasenetsky Krasnoyarsk State Medical University, 660022 Krasnoyarsk, Russia; katya_n2001@mail.ru (E.A.N.); stk99@yandex.ru (M.M.P.); irinavr2018@gmail.com (I.V.R.); 4 Department of Neurogenerative Disorders, Yakut Science Centre of Complex Medical Problems, 677000 Yakutsk, Russia; tanya.davydova.56@inbox.ru; 5 Hospital for War Veterans, 193079 Saint Petersburg, Russia; vera.v.trefilova@yandex.ru; 6 Department of Therapy and General Medical Practice with a Course of Postgraduate Professional Education, Altai State Medical University, 656038 Barnaul, Russia; g.a.chumakova@mail.ru

**Keywords:** amyotrophic lateral sclerosis, syringomyelia, Chiari 1 malformation, cerebrospinal fluid, related disorder, differential diagnosis, adult, ALS mimic syndrome

## Abstract

Chiari 1 Malformation (CM1) is classically defined as a caudal displacement of the cerebellar tonsils through the foramen magnum into the spinal cord. Modern imaging techniques and experimental studies disclose a different etiology for the development of CM1, but the main etiology factor is a structural defect in the skull as a deformity or partial reduction, which push down the lower part of the brain and cause the cerebellum to compress into the spinal canal. CM1 is classified as a rare disease. CM1 can present with a wide variety of symptoms, also non-specific, with consequent controversies on diagnosis and surgical decision-making, particularly in asymptomatic or minimally symptomatic. Other disorders, such as syringomyelia (Syr), hydrocephalus, and craniocervical instability can be associated at the time of the diagnosis or appear secondarily. Therefore, CM1-related Syr is defined as a single or multiple fluid-filled cavities within the spinal cord and/or the bulb. A rare CM1-related disorder is syndrome of lateral amyotrophic sclerosis (ALS mimic syndrome). We present a unique clinical case of ALS mimic syndrome in a young man with CM1 and a huge singular syringomyelic cyst with a length from segment C2 to Th12. At the same time, the clinical picture showed upper hypotonic-atrophic paraparesis in the absence of motor disorders in the lower extremities. Interestingly, this patient did not have a disorder of superficial and deep types of sensitivity. This made it difficult to diagnose CM1. For a long time, the patient’s symptoms were regarded as a manifestation of ALS, as an independent neurological disease, and not as a related disorder of CM1. Surgical treatment for CM1 was not effective, but it allowed to stabilize the course of CM1-related ALS mimic syndrome over the next two years.

## 1. Introduction

Chiari malformation type 1 (CM1) is characterized by the caudal descent of the cerebellar tonsils through the foramen magnum at least 5 mm in adults, measured using the McRae line drawn from the basion to the opisthion [1,2,3]. Nearly 33% of patients with typical symptoms of CM1 had tonsillar ectopia from 2 to 4 mm [4]. According to the definition of the Chiari Consensus Conference held in Milan in 2019, CM1 was considered herniation of one or both cerebellar tonsils ≥ 5 mm below the McRae line or even 3 to 5 mm but with syringomyelia (Syr) or peg-like appearance of the tonsils [5]. Even though CM1 is classified as a rare disease (ORPHA268882), CM1 has been diagnosed in up to 1–3.6% of the population using magnetic resonance imaging (MRI) and symptomatically affects 0.1% of the general population [6].

CM1 can present with a wide variety of abnormalities such as hydrocephalus [7], spina bifida [8], spinal deformity [9], tethered cord syndrome [10], craniosynostosis [11], Ehlers-Danlos syndromes [12], Klippel-Feil syndrome [13], and Syr [3,4,14]. The association between CM1 and Syr varies from 20 to 74% [15,16,17]. In this regard, the International Classification of Diseases of 11 revision (ICD-11, 2018) classifies CM1 as follows: Arnold-Chiari syndrome without spina bifida or hydrocephalus (Q07.00); Arnold-Chiari syndrome with spina bifida (Q07.01); Arnold-Chiari syndrome with hydrocephalus (Q07.02); and Arnold-Chiari syndrome with spina bifida and hydrocephalus (Q07.03) [18]. According to the diagnostic recommendations of the Interregional Chiari and Syringomyelia Consortium, CM1 is classified into CMI-A, in the presence of Syr on magnetic resonance imaging (MRI) and CMI-B, in the absence of Syr on MRI [16].

Syr is considered as a chronic progressive disorder that is characterized by the development of fluid-filled longitudinal cavitation (syrinxes) inside the spinal cord. The spinal cord cavities are filled with cerebrospinal fluid (CSF) or a fluid similar in composition to it [19]. Syr is a rare disease (ORPHA3280); the prevalence varies from 1.9 to 8.4 per 100,000 population [20,21]. The widespread use of MRI has increased the number of cases diagnosed with Syr [22,23,24]. For example, the diagnosed cases of Syr increased by 9.78 times between 1971 and 2003 years in New Zealand [20]. This increase of Syr prevalence is largely due to the accidental detection of Syr signs in the study of patients with spinal pain using MRI [25]. Syr is diagnosed two times more often in adults (especially in the third decade of life) than in children. The frequency of symptomatic forms of Syr is higher in adults than in children (40% versus 23%, respectively) [26]. This fact is explained by precipitating factors that contribute to an increase in CSF pulse pressure at the craniovertebral junction as microtrauma and physical activity, which result in the accumulation of changes in the spinal cord with age [27]. Females are affected more often than males by 17–27% [28]. Sensory disorders are diagnosed in 48% of cases, and motor disorders in 32% cases of CM1-related Syr [16]. At the same time, the frequency of asymptomatic CM1-related Syr varies from 7% to 40% [15,16].

There is growing scientific and clinical interest in Syr-associated amyotrophic lateral syndrome (also known as ALS mimic syndrome) among all Syr-related disorders in patients with CM1 [4,6,13,29], because it requires a differential diagnosis with idiopathic ALS (also known as Lou Gehrig’s disease) [30]. ALS is characterized by damage to the motor neurons of the anterior horns of the spinal cord with the formation of progressive muscle atrophies and fasciculations, which can outpace the development of motor deficit [31]. Syr-associated ALS mimic syndrome is caused by mechanical compression of the anterior horns of the spinal cord by a syrinx, unlike idiopathic ALS [32]. In consequence, the treatment strategies and prognosis differ significantly between Syr-associated ALS mimic syndrome and idiopathic ALS. At the same time, the early diagnosis of these disorders can help stop or slow down its progression and thereby improve the prognosis. For unknown reasons, some patients with CM1-related Syr develop anterior horn lesions without degenerative changes in other neural structures adjacent to the syrinx. As a result, damage to the lower motor neuron (LMN) develops without damage to the upper motor neuron (UMN). This form of ALS mimic syndrome may be associated with a reduced resistance of spinal motoneurons to hypoxia and compression compared to fibers of the pyramidal tracts in the lateral and anterior cortical-spinal tracts. However, Syr-associated ALS mimic syndrome is extremely rare in real clinical practice, which was the reason to present our evidence-based clinical case study of this disorder in a young Caucasian male.

## 2. Case Presentation

### 2.1. Patient History

A Caucasian male, 23 years old, married with 2 children. He is a bus driver. This patient has been under our observation for 4 years. He complained on a slight weakness, muscle wasting of both hands, loss of dexterity, and clumsiness with fine motor skills. The patient has no family history of CM1, Syr, and ALS. The age of onset was 19 years, when the patient first noticed a slight weakness when squeezing the right hand. Weakness in his arms gradually progressed. After 2 years, the patient showed progressive loss of muscle mass (hypotrophy) of the thenar, hypothenar and dorsal interosseous muscles of the right hand. However, these symptoms did not particularly prevent him from driving the bus. CM1-related Syr was first diagnosed at the age of 19 years after cervical spine MRI examination was ordered by a neurologist to exclude herniated disc and radiculopathy. At the age of 20 years, Syr-associated ALS mimic syndrome was first diagnosed based on neurological symptoms, as well as electromyography (EMG) and MRI findings. At the age of 22 years, the patient underwent suboccipital craniotomy with C1 laminectomy. After this operation, there was no significant increase in muscle weakness and atrophy of the muscles of the hands during the last year. Despite the progression of the disease, the patient is socially and professionally adapted to the present time.

### 2.2. Physical Examination

Consciousness is clear. The patient is oriented in his own personality, place, time, and space. Emotional lability is manifested by the inability to control the expression of emotions, but there are no cognitive impairments. Cranial nerves are normal.

**Figure 1:**
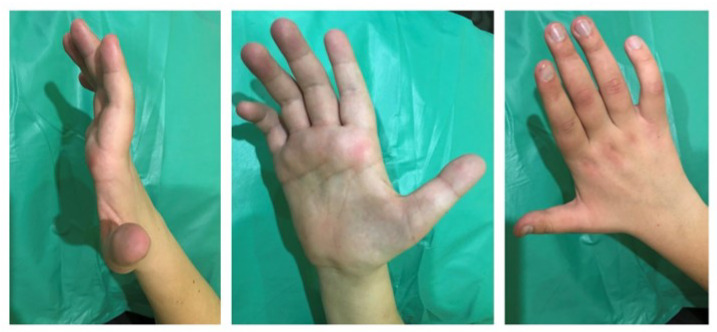
Mild flexion deformity of the fingers and hypotrophy of the muscles of the right hand in a 23-year-old man with Chiari malformation type 1-related syringomyelia.

A slight flexion deformity of the fingers of the hands (predominant on the right hand) was detected (Figure 1). There is moderate hypotrophy of the muscles of both hands; it is more pronounced on the right. There are spontaneous periodic fasciculations of the muscles of the thenar and hypothenar of both hands (more on the left), intensified after a hammer percussion of muscle. Less pronounced spontaneous and induced fasciculations of the muscles of the shoulders and forearms were detected on both sides (more on the right).

Thumb opposition strength decreased up to 2/5 points on the right hand and up to 4/5 points on the left hand. Metacarpophalangeal abduction strength of the fingers reduced up to 3/5 points on the right hand and up to 4/5 points on the left hand. Flexion strength of the second, third, fourth, and fifth fingers decreased up to 2/5 points on the right hand and up to 4/5 points on the left hand. Flexion and extension of the wrist slightly reduced on the right hand up to 4/5 points. The strength of the muscles of the forearms, shoulders, upper arm, neck, trunk, and lower extremities were normal (5/5 points).

Carporadial, flexion-elbow, and extensor-elbow reflexes are hypoactive bilaterally with left laterality. Bekhterev bone-abdominal reflex, deep abdominal reflex, Bekhterev scapulohumeral reflex, and knee and Achilles reflexes were normal on both sides (Table 1). The pathological reflexes Babinski, Chaddock, Oppenheim, Gordon, and Schaefer were absent. No coordination disorders were identified.

Touch, vibration, temperature, position, and pain sensations were normal. There are no violations of defecation, urination, and erectile function.

### 2.3. Laboratory Results

Routine biochemical and hematologic study results were normal, including serum levels of 25OH(D_3_), cyanocobalamin, thyroid hormones (T3, T4, thyroid—stimulating hormone), glycated hemoglobin, and creatine kinase. The serologic testing results for hepatitis B and C, human immunodeficiency virus, and Treponema pallidum were unremarkable.

### 2.4. Neuroimaging

**Figure 2:**
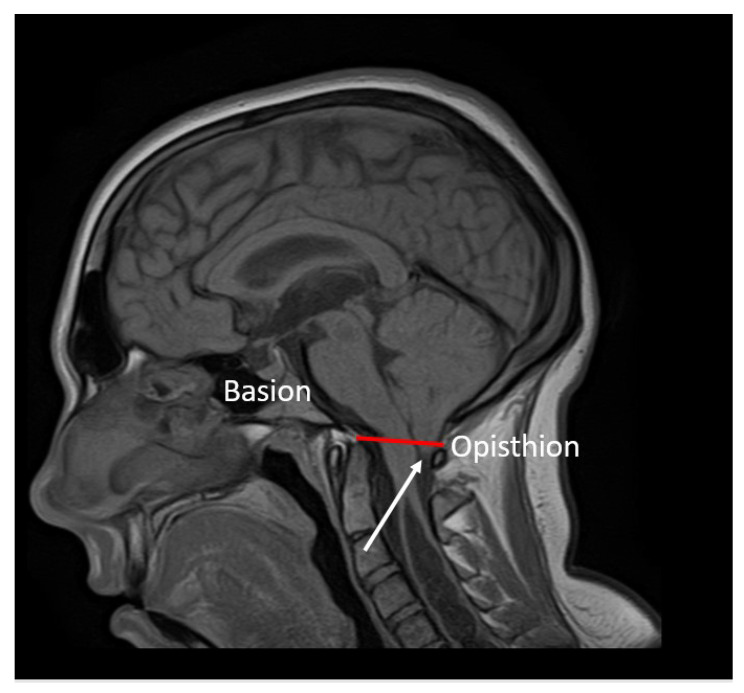
Sagittal T1-weighted MRI scans of the brain: caudal descent of the cerebellar tonsils 6 mm (white arrow) below the McRae line (red line).

Brain MRI: T1- and T2-weighted MRI scans revealed cerebellar tonsillar herniation of more than 6 mm below the foramen magnum (Figure 2). This allowed us to diagnose CMI in the patient.

**Figure 3:**
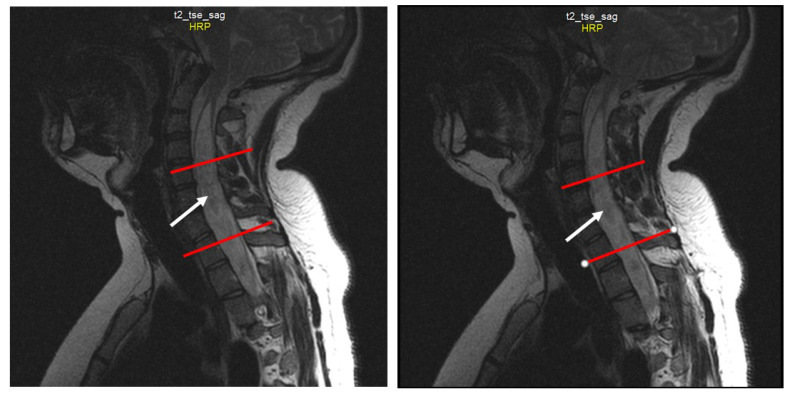
Sequential sagittal T2-weighted MRI scans of the cervical spine demonstrate the syrinx (white arrow) from the level of C2 extending inferiorly into the thoracic spinal cord, with the greatest dilation around C5-Th1 (between the red lines).

**Figure 4:**
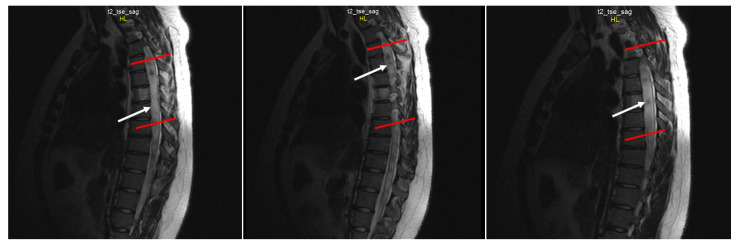
Sequential sagittal T2-weighted MRI scans of the thoracic spine demonstrate the syrinx (white arrow) extending inferiorly down to the Th12 level with the greatest dilation around Th3-Th7 (between the red lines).

**Figure 5:**
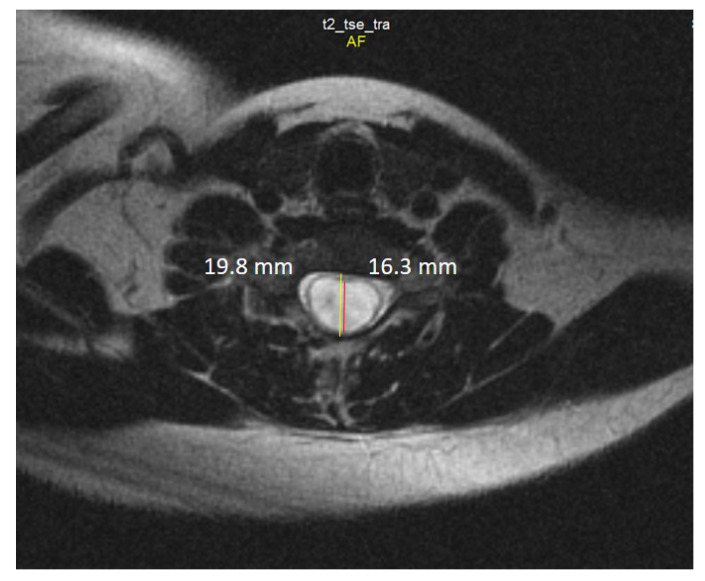
Axial T2-weighted MRI scan of the cervical spine demonstrates anteroposterior dilation of the syrinx (red line) and spinal cord (yellow line) at the level of the C5 vertebra.

**Figure 6:**
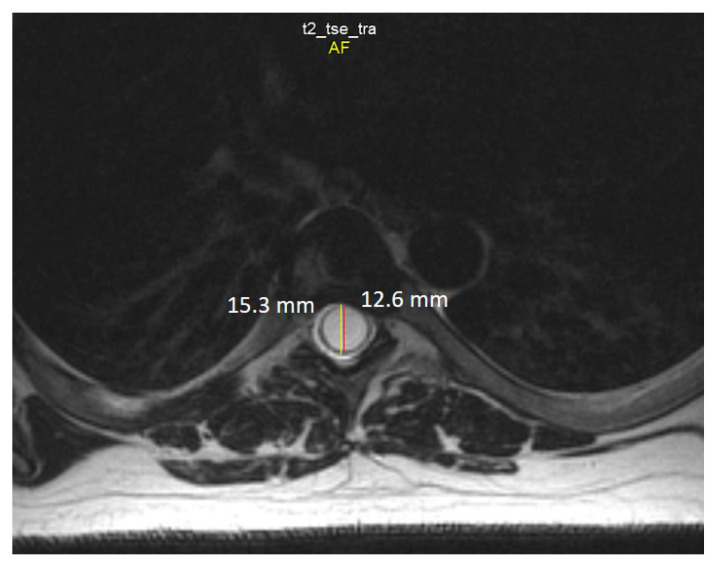
Axial T2-weighted MRI scan of the thoracic spine demonstrates the anteroposterior dilation of the syrinx (red line) and spinal cord (yellow line) at the level of the Th6 vertebra.

Spine MRI: The radiological survey of the cervical and thoracic spine showed no abnormalities. However, T1- and T2-weighted MRI scans of the cervical and thoracic spine showed a large singular syrinx, spreading in the cervicothoracic cord from C2 to Th12. The syrinx is more expanded between the cervical segments of C5 and Th1 (Figure 3) and between thoracic segments of Th3 and Th7 (Figure 4). Anteroposterior diameter of the syrinx at C5 was 16.3 mm (Figure 5) and at Th6 was 12.6 mm (Figure 6), while the anteroposterior diameters of the spinal cord at C5 and Th 6 were 19.8 and 15.3 mm, respectively.

**Figure 7:**
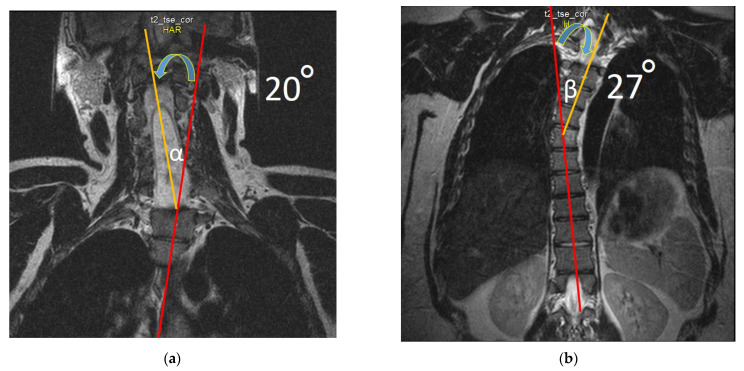
T2-weighted MRI scan of the cervical (**a**) and thoracic spine (**b**) demonstrate scoliosis at the cervicothoracic level. Note: Red lines are drawn along the axis of the spine; yellow lines are drawn by the deviated part of the spine from the axis of the spine; blue arrows indicate the direction of curvature. In degrees, deflection angle in the cervical region (α) and in the thoracic region (β) is determined.

In addition to changes in the structure of the spinal cord, coronal sections revealed signs of moderate curve ‘S’-shaped scoliosis. In the thoracic region, the scoliotic curve bends to the left by 27 degrees, and in the cervical region, the scoliotic curve bends to the right by 20 degrees (Figure 7), which is a characteristic feature for Syr. As we know, the development of scoliosis in such patients may be associated with denervation of the paraspinal muscles [33,34].

### 2.5. Electrophysiology

#### 2.5.1. Evoked Electromyography

**Figure 8:**
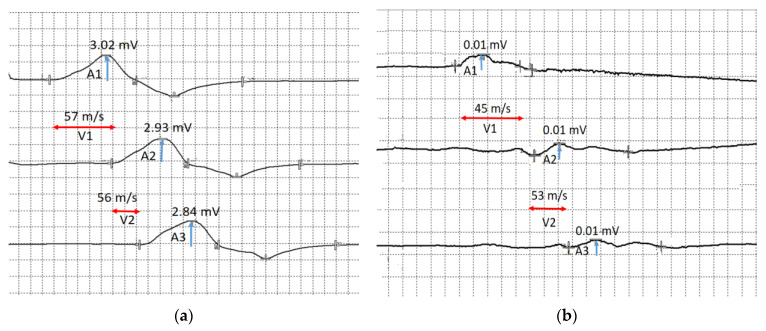
The motor conduction velocity of the ulnar nerves: (**a**) motor conduction velocity of the left ulnar nerve below the elbow (V1) and above the elbow (V2), and the compound muscle action potential in response to stimulation at the wrist (A1), below the elbow (A2) and above the elbow (A3); (**b**) motor conduction velocity of the right ulnar nerve below the elbow (V1) and above the elbow (V2), and the compound muscle action potential in response to stimulation at the wrist (A1), below the elbow (A2) and above the elbow (A3). Blue arrows show compound muscle action potential amplitude and red arrows show conduction velocity.

In the upper extremities: motor and sensory nerve conduction and various F-response parameters have been examined in the median and ulnar nerves bilaterally. Distal latency and motor conduction velocity in the median and ulnar nerves were normal. Pronounced decrease of the compound muscle action potential (CMAP) was recorded on examination of the right median and ulnar nerves to 0.09 mV and 0.01 mV, respectively, and moderate decrease of the CMAP was registered on examination of the left ulnar nerve to 3.0 mV (Figure 8). The CMAP of the left median nerve was normal (CMAP > 5 mV).

**Figure 9:**
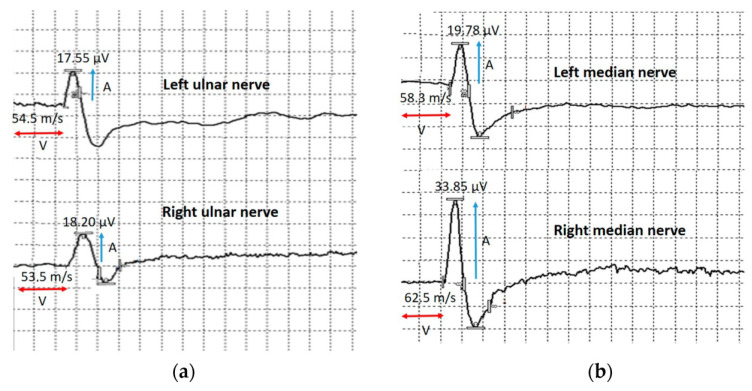
Sensory conduction velocity of the upper extremities nerves. (**a**) Sensory conduction velocity (V) and sensory nerve action potential amplitude of the left and right ulnar nerves. (**b**) Sensory conduction velocity (V) and sensory nerve action potential amplitude (A) of the left and right median nerves. Blue arrows show compound muscle action potential amplitude and red arrows show conduction velocity.

Sensory nerve conduction velocity and sensory nerve action potential (SNAP) amplitude in the bilateral median and ulnar nerves were normal (Figure 9).

In the lower extremities: motor and sensory nerve conduction velocities, CMAP amplitude, SNAP amplitude and various F-response parameters of the bilateral peroneal and tibial nerves were normal. Sural nerves studies showed normal conduction velocities and normal SNAP amplitude bilaterally.

#### 2.5.2. Quantitative Needle Electromyography

In the upper extremities, quantitative needle EMG was performed in the deltoid muscle (n. axillaris, C5-Th1), biceps brachii (n. musculocutaneus, C5–C7), brachioradialis (n. radialis, C5–C7), first dorsal interosseous muscle of the hand (n. ulnaris, C8—Th1), abductor digiti minimi (n. ulnaris, C7–Th1), and abductor pollicis brevis muscle (n. medianus, C6–C7) bilaterally.

**Figure 10:**
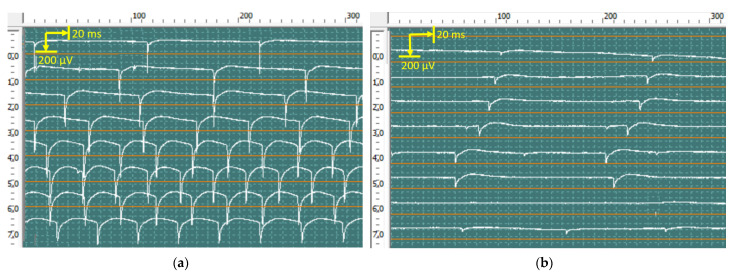
Recorded positive sharp waves from the first dorsal interosseous muscle of the left (**a**) and right (**b**) hands via concentric needles. The amplitude of positive sharp waves was 850 μV in the right hand and 350 μV in the left hand.

**Figure 11:**
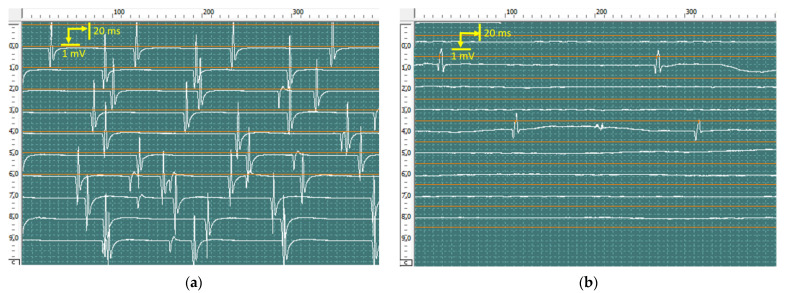
Recorded fasciculation potentials from the abductor digiti minimi of the left (**a**) and right (**b**) hands using concentric needles. The amplitude of fasciculation reached 6120 μV in the left hand and 1540 μV in the right hand.

At rest, pronounced spontaneous activity was registered, such as fibrillation potentials, positive sharp waves (Figure 10), and fasciculation potentials (Figure 11) in the right abductor pollicis brevis muscle, bilateral abductor digiti minimi, and bilateral first dorsal interosseous muscle of the hand. Rare fibrillation, positive sharp waves, and fasciculation potentials waves were registered on bilateral examination of the deltoid muscle, biceps brachii muscle, and brachioradialis muscle and in the left abductor pollicis brevis muscle. In muscles, the f hands firing rate, frequency, and amplitude of positive sharp waves and fasciculation potentials were markedly higher on the left side.

Motor unit potential (MUP) analysis at slight voluntary muscle contraction revealed a significant increase in amplitude, duration, and size index of the MUP in the right abductor pollicis brevis muscle, right abductor digiti minimi, and right first dorsal interosseous muscle of the hand. MUP recruitment markedly reduced. The index of polyphasic MUP averaged 65%. In the left abductor digiti minimi and left first dorsal interosseous muscle of the hand, the amplitude, duration, and size index of the MUP were slightly enlarged, MUP recruitment was normal, and 25% polyphasic MUP was recorded. No significant changes were found on bilateral examination of the deltoid muscle, biceps brachii muscle, and brachioradialis muscle.

These changes suggest neurogenic abnormalities such as chronic denervation and re-innervation with acute denervation activity in the right abductor pollicis brevis muscle, bilateral abductor digiti minimi, and bilateral first dorsal interosseous muscle of the hand. Results indicate damage to the anterior horns of the spinal cord at the level of the C6-Th1 segments with loss of motor axons of the right median and ulnar nerves.

Quantitative needle EMG was performed in the tibial anterior, gastrocnemius, and vastus lateral muscles in the lower extremities bilaterally. These findings suggest the absence of abnormality in the investigated nerves and muscles of lower extremities and in the anterior horns of the spinal cord at the level of the L1-S2 segments.

Moreover, we examined the spontaneous activity in the tongue and rectus abdominal muscle. We did not record any fibrillation potentials, positive sharp waves, or fasciculation potentials at all.

## 3. Discussion

A 23-year-old Caucasian male with no significant past medical history presented to the Clinic of Brain and Vertebral Diseases (Moscow region, Russia) with atrophy and weakness of the small muscles of the right hand and moderate weakness of the left wrist. Symptoms appeared 4 years ago with initial weakness and hypotrophy in the right hand with a gradual increase in weakness in the left hand without any sensory loss. Moderate claw hand deformity of the right hand had developed. Bilateral fasciculations of the hands were visualized. Due to these symptoms, ALS was suspected. Impairment of the UMN was excluded, because the patient had no pathological reflexes, and periosteal and deep tendon reflexes in the upper and lower extremities were normal (except mild decrease of brachioradialis, elbow flexion, and triceps reflexes).

Findings on brain MRI revealed that the cerebellar tonsillar descended by 6 mm under the Mc-Rae line, which is typical for CM1. On spinal MRI, a syringomyelia was observed, which was formed by one huge singular syrinx. The syrinx originated from the C2 vertebra to the Th12 vertebra. The anteroposterior diameter of the syrinx had a maximum dilation at the C5–C7 and Th3–Th7 levels and reached 16.3 mm and 12.6 mm, respectively. Based on the clinical, neurophysiological, and MRI investigations, we came to the conclusion that neurological disorders were due to impairment of the anterior horns of the spinal cord at the C6-Th1 level. The impairment developed as a result of chronic motoneuron compression by the dilated syrinx at this region. The presence of CM1 on brain MRI was the basis for considering CM1-related Syr.

Neurophysiological findings of evoked EMG and conventional needle EMG were most likely due to damage to the anterior horns of the spinal cord at the C6-Th1 level predominantly in the right side.

Therefore, the differential diagnosis of Syr-associated ASL mimic syndrome in the patient with CM1 was carried out with other disorders (Table 2).

On differential diagnosis, cervical myelopathy was excluded due to the absence of impairment of the UMN in the lower extremities. Cervical spondylotic amyotrophy was excluded because of the absence of radicular compression on cervical spine MRI. Muscular atrophy in clinical cases did not appear similar to oblique amyotrophy associated with Hirayama disease. In addition, normal T2-weighted signals of the spinal cord on MRI at the site of the maximum forward shift in the cervical level led to the exclusion of Hirayama disease.

The absence of conduction blocks of motor nerves in EMG and correlated weakness with severity of muscle atrophy were the reason to exclude multifocal motor neuropathy.

According to the El Escorial criteria, the evidence of lower motor neuron degeneration on clinical and neurophysiological examination of the cervical region and progressive spread of symptoms within one region, but not to other regions, was not enough to diagnose ALS. That is because the presence of clinical disorders of the upper motor neurons is the minimum requirement to diagnose possible ALS [43]. The flail arm syndrome and idiopathic ALS were excluded.

As a result, on the basis of the MRI findings and characteristic changes in evoked and needle EMG, Syr-associated ALS mimic syndrome with CM1 was diagnosed in our patient.

We searched for similar clinical cases in the literature using the PubMed-indexed journals search system. The criteria of search were as follows: Syringomyelia, Chiari Malformation I Type, an Amyotrophic lateral sclerosis. Among 2353 studies, 12 studies about 17 cases met the eligibility criteria (Table 3).

Thus, the lesion of only the LMN was demonstrated by the authors in just 7 cases. This indicates the extreme rarity of the development of isolated LMN lesions, without UMN impairment, in Syr-associated ALS mimic syndrome related with CM1. In our opinion, the predominant lesion of the anterior horns of the spinal cord because of compression by a syringomyelic cyst in our patient is most likely associated with a decrease in the resistance of these structures to compression and ischemia compared to the surrounding spinal cord tissue. In addition, the absence of a lesion of the UMN on an examination of the lower extremities indicates a high plasticity of the pyramidal pathways, despite pronounced syringomyelic changes in the spinal cord throughout between the C2 vertebra to the Th12 vertebra.

The nature of the work may be related to risk factors for the development of symptoms of Syr-associated ALS related with CM1, as the driver of a vehicle such as a bus is subjected to vibration and shaking for 6 hours 5 days a week. Obviously, prolonged and frequent mechanical stress on the spine and cranio-vertebral junction may adversely affect the morphological, neurophysiological, and clinical manifestations of Syr and ALS in patients with CM1. On the other hand, clinical cases of CM1-related ALS among bus drivers are not common in the literature [56].

## 4. Conclusions

CM1-related Syr may be the cause of the development of ALS mimic syndrome as a result of prolonged compression and ischemia of the anterior horns of the spinal cord. Our clinical case demonstrates that ALS mimic syndrome may be rare clinical disorder in patients with CM1-related Syr, while the characteristic symptoms for Syr are clinically absent or minimal.

A complex approach is needed to diagnose these disorders, including MRI of the head and spine, as well as induced evoked and needle EMG.

## 5. Declaration of Patient Consent

The authors confirm that they have obtained all necessary patient consent forms. In the form, the patients gave their consent for the publication of their images and other clinical information in the journal. The patients understand that their names and initials will not be published and appropriate steps will be taken to conceal their identity, but anonymity cannot be guaranteed.

## Figures and Tables

**Figure 12:**
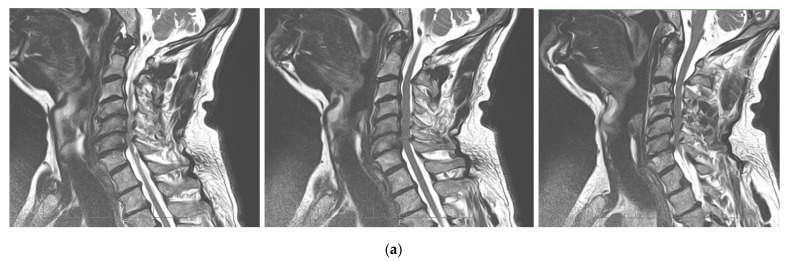

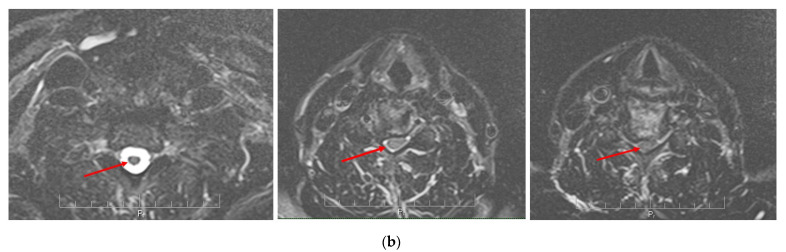
Sequential T2-weighted MRI sagittal scans (**a**) and axial scans (**b**) of the cervical spine demonstrate compression of the spinal cord (red arrow) via spondylotic changes and herniated discs at the level of C3–C7 in a 65-year-old patient with cervical myelopathy.

**Table 1:** Tendon and periosteal reflexes.

Reflex	Left	Right	Comparison between the Parties
Brow reflex	Normal	Normal	D = S
Mandibular reflex	Normal	Normal	D = S
Flexion-elbow reflex	Decreased	Decreased	D < S
Carporadial reflex	Decreased	Decreased	D < S
Bekhterev scapulohumeral reflex	Normal	Normal	D = S
Extensor-elbow reflex	Decreased	Decreased	D < S
Bekhterev bone-abdominal reflex	Normal	Normal	D = S
Deep abdominal reflex	Normal	Normal	D = S
Knee reflex	Normal	Normal	D = S
Achilles reflex	Normal	Normal	D = S

**Table 2:** Differential diagnosis of syringomyelia-associated amyotrophic lateral sclerosis mimic syndrome in the patient with Chiari malformation type 1.

Disorder	Clinical Findings	MRI Findings	Pathogenesis
Cervical myelopathy [35]	Neck pain, hand numbness and paresthesia, loss of fine dexterity, amyotrophy and weakness of both the upper and the lower limbs, gait difficulties, Lhermitte’s phenomenon, and sphincter incontinence.	Intramedullary signal abnormalities due to edema and structural changes with a hyperintense signal on T2-weighted MRI (Figure 12)	Compression of the spinal cord by spondylotic changes.
Cervical spondylotic amyotrophy [36]	Slowly progressive amyotrophy with weakness of the proximal extremity of one or both upper limbs, often preceded by a transient period of shoulder pain without sensory loss	Narrowed intervertebral space between the vertebras of the cervical level. Sagittal T2-weighted magnetic resonance image showing root compression and impingement against prolapsed cervical discs	Isolated intradural compression of multiple motor roots, without damage to either the spinal cord or the sensory roots.
Hirayama disease [37]	Predominantly unilateral upper extremity weakness and atrophy, cold paresis, and no sensory or pyramidal tract involvement. It is also characterized by muscle weakness and atrophy in the hand and forearm with sparing of the brachioradialis, giving the characteristic appearance of oblique amyotrophy that affects the C7, C8 and T1 myotomes. The amyotrophy is unilateral in most patients, asymmetrically bilateral in some and rarely symmetric.	Abnormal T2-weighted signal of the spinal cord at the site of maximum forward shift without an obvious cause neutral position: abnormal T2-weighted signal of the spinal cord at the site of maximum forward shift without an obvious cause.	Spinal cord compression by the posterior dural sac during neck flexion
Multifocal motor neuropathy [38,39]	The pattern of weakness may be related to specific nerves. Due to the development of paresis as a result of conduction block, a high degree of weakness does not correlate with moderate muscle atrophy. Sensory disturbances could not be detected. Often recognized in patients with a short disease duration. In EMG complex unstable MUPs without spontaneous activity at rest. Rapid recruitment pattern disproportionate to muscular atrophy. Fasciculation potentials may be recoded.	No specific changes on MRI	Chronic progressive immune-mediated motor neuropathy, which leads to progressive asymmetric weakness and partial motor conduction block in EMG. Anti-GM1 antibodies are identified in at least 40% of patients.
Idiopathic ALS [40,41]	Progression of lower motor neuron signs (including EMG features in clinically unaffected muscles) and upper motor neuron signs, muscle atrophy, paralysis, frontotemporal dementia with absence of sensory signs. EMG is characterized by fasciculations in one or more regions, neurogenic changes, normal motor and sensory nerve conduction and absence of conduction blocks.	Bilateral symmetric T2 and FLAIR hyperintensities of the corticospinal tract. Decrease of cerebral volume in the gray matter of the frontal and temporal lobes. Gliosis and axonal degeneration of the anterolateral spinal cord, manifested by T1 hyperintensity and T2 hyperintensity.	The pathogenesis of ALS is still unknown. Mitochondrial dysfunction in cells leads to serious disorders associated with pathological changes in ALS, such as disturbances in the calcium buffer period, excessive formation of free radicals, which leads to an increase in mitochondrial membrane permeability and oxidative stress.
The flail arm syndrome [42]	Slow progressive, predominantly proximal weakness, and atrophy of the upper limbs. Symptoms may be limited to the cervical region without functional involvement of the lower extremities, pectorals, or bulbar muscles for at least 12 months. Five patients out of 10 develop signs of the UMN in the upper extremities and 7 patients out of 10 in the lower extremities.	Same as ALS	Involvement of the corticospinal tract in Flail arm syndrome is identical to that in the patients with ‘classic’ ALS. Flail arm syndrome had a pattern of microstructural alterations in corticofugal tracts identical to those seen in ALS. Because of that there is a hypothesis that flail arm syndrome is such a phenotype of ALS.

Note: ALS—amyotrophic lateral sclerosis; EMG—electromyography; FLAIR—fluid-attenuated inversion recovery; GM1—ganglioside-monosialic acid; UMN—upper motor neuron; MUP—motor neuron potential.

**Table 3:** Clinical cases of Syr-associated amyotrophic lateral sclerosis mimic syndrome in patients with Chiari malformation type 1 in the PubMed database.

Author	Year	Sex	M/Age	Cl/ Age	Upper Extremities	Lower Extremities	EMG	**Initial Side**	**Asymmetry**	**MNI**
Petit et al. [44]	1984	M	24	25	Weakness, amyotrophy, fasciculations and areflexia were found in the upper limbs without sensory disorders.	Hyperreflexia in the lower limbs.		Both sides	S = D	UMN LMN
Lim et al. [45]	1989	F	30	40	Progressive weakness and hypotrophy of the upper limbs with hypotonia and areflexia were noted especially in the left side. Strength in the proximal and distal muscles generally was 2/5 points in the right hand and 1/5 points in the left hand. Dissociated sensory loss to pain and temperature was over C4 to T1 dermatomes.	Weakness and spasticity in the lower limbs. Strength in the flexor muscles was 4/5 points.		Both sides	S > D	LMN UMN
Lim et al. [45]	1989	F	37	43	The first symptom was numbness, followed by weakness in the left upper extremity and then in the right upper extremity Subsequently, the numbness progressed to involve the chest anteriorly and posteriorly. The upper limbs were hypotonic and areflexic. Strength in the hands was 1/5 point on the left and 3/5 points on the right. There was diminished sensation of all modalities including the vibration and position senses but especially the pain and temperature from left C5 to L1 dermatomes	Spasticity and weakness in the lower limbs. Strength was 4/5 points.		Left side	S > D	LMN UMN
Lim et al. [45]	1989	F	42	62	Right-sided ptosis was diagnosed at the age of 42 years. At age of 62 years, hypotrophy, weakness, and numbness of the upper extremities first developed in the right hand and then spread to the left with hypotonia and hyporeflexia. Strength in the proximal muscles was 3/5 points and 2/5 points in the distal muscles.	Spasticity and mild weakness in the lower extremities.		Right side	S = D	LMN UMN
Lim et al. [45]	1989	M	26	27	Hypotrophy and weakness of both hands progressively developed. Hypotonia and hyporeflexia were observed only in the right hand. Sensory disorders began with a painless burning feeling over the left forearm. Later, progressive numbness developed in the left side of the neck, left upper limb, right upper limb, and spread to the left side of the trunk down to the middle of the left thigh. Loss of pain and temperature sensation with the preservation of touch; position and vibration senses were found over the left C2 to L2 and over the right C2 to Th7	Mild spasticity and weakness of the lower extremity.		Both sides	D > S	LMN UMN
Hamada et al. [46]	1990	M	54	59	Weakness and atrophy of the proximal muscles of both the upper extremities at the age of 56 years. Hyperreflexia in the upper extremities.	Weakness of both the lower extremities started at the age of 54 years. Hyperreflexia and respiratory muscle weakness, pathological reflexes on both legs, and tongue fasciculation developed successively within 5 years before death from distress at 59 years of age.		Both sides	S = D	LMN UMN
Yoshitoshi et al. [47]	1992	F	39	43	Muscle weakness and atrophy were prominent in the bilateral deltoid muscles. No hypotrophy and weakness signs were noted in the bilateral forearms and hand muscle. There was no sign of sensory impairment except vibratory sensation.	Normal	Neurogenic disorder of motor unit potential in EMG in the deltoid muscles.	Both sides	S = D	LMN
Titlic et al. [48]	2008	F	42	44	Left hand Weakness in 4–5 fingers. Hypoesthesia in C4–C8 segments	Normal	EMG indicates a moderate chronic radicular bilateral lesion of the C7 root and an initial right-side lesion of the C6 and C8 roots	Left side		LMN
Cağan et al. [49]	2010				Weakness of the upper extremities with decrease of strength up to 1/5 point bilaterally.	Normal	EMG revealed bilateral brachial plexus palsy.	Both sides	S = D	LMN
Waqar et al. [50]	2013	F	16	21	Slight weakness in the hands and hypotrophy of the thenar and hypothenar eminences of the left hand. Numbness and pain in the upper limbs and loss of pain and temperature sensation in the C3-T2 dermatomes, bilaterally.	Normal		Left side	S > D	LMN
Kadoya et al. [51]	2015		40	40 (2 Months)	Numbness and muscular weakness of the bilateral upper limbs.	Normal		Both sides	S = D	LMN
Kadoya et al. [51]	2015		32	32 (5 Months)	Muscular weakness of the bilateral upper limbs	Numbness and muscular weakness of the lower limbs developed for 2 months.		Both sides	S = D	LMN UMN
Mora et al. [52]	2015	M	16	55	At the age of 16 years, weakness was observed in the right hand. At 55 years old, the patient was diagnosed with asymmetric focal segmental atrophy of his bilateral forearm flexor and extensor muscle groups with preservation of the bilateral brachioradialis muscles. Strength in the proximal muscles was 4/5 to 5/5 points and 1/5 point in the distal muscles.	Mild weakness. Strength in the left hip extension and flexion was 4/5 points and 4/5 points in the bilateral knee extensors.	Absence of CMAP of his left median, ulnar, and radial nerves. SNAP responses were normal and slightly low in the ulnar and radial nerves. CMAP of the peroneal and tibial nerves and SNAP of sural nerves were normal. SNAPs were normal. EMG of his upper extremities demonstrated evidence of diffuse chronic neurogenic changes in the C5 to T1 innervated muscles as well as evidence of active denervation in his right triceps brachii. In his left lower extremity, chronic neurogenic changes were noted in the gastrocnemius medial head. His left cervical paraspinal muscles were normal on EMG.	Left side	S > D	LMN UMN
Lan et al. [53]	2017	M	21	44	Severe weakness: Strength in the proximal muscles was 0/5 points and 0/5 points in the distal muscles. Severe atrophy of the bilateral forearm flexor and extensor muscle groups.	Normal	Absence of CMAP of the median, ulnar, and radial nerves with slightly abnormal SNAP of the median, ulnar, and radial nerves. EMG of all nerves in the lower extremities was normal. All the nerves on the lower limbs have normal CMAPs and normal SNAPs.	Both sides	S = D	LMN
Storti et al. [54]	2021	M	20	42	Distal and proximal weakness and hypotrophy in the hands developed and progressed slowly to all four limbs especially on the left side for 20 years. Bilateral areflexia of the upper extremities was noted.	Spasticity, hyperreflexia, pathologic positive Babinski sign bilaterally and clonus in the lower extremities	EMG, performed 3 years later, was consistent with a diagnosis of definite ALS.	Both sides	S > D	LMN UMN
Zheng et al. [55]	2022	F	55	73	Mild weakness of the arms. Sensory loss to pain and temperature in the whole body sparing the face.	Mild weakness in the lower extremities		Both sides	S = D	LMN UMN
Zheng et al. [55]	2022	M	18	60	Atrophy and weakness of the shoulder abduction and intrinsic hand muscles more on the left hand than in the right hand.	Normal	EMG studies showed chronic denervation–reinnervation in C5 to C7 muscles bilaterally and in C8 muscles on the left	Left side	S > D	LMN

Note: F—female; M—male; M/age—manifestation age; Cl/age—clinical examination age; S—left side, R—right side; ALS—amyotrophic lateral sclerosis; EMG—electromyography; LMN—lower motor neuron; UMN—upper motor neuron; MNI—motor neuron impairment; CMAPs—compound muscle action potentials; SNAPs—sensory nerve action potentials.

## Data Availability

Not applicable.
